# Diagnostic finding on high-resolution computed tomography (HRCT) predicts a good response to pirfenidone in patients with idiopathic pulmonary fibrosis

**DOI:** 10.1097/MD.0000000000033722

**Published:** 2023-05-12

**Authors:** Miao Ma, Min Cao, Yujuan Gao, Xiaohua Qiu, Hanyi Jiang, Hourong Cai

**Affiliations:** a Department of Respiratory and Critical Care Medicine, Nanjing Drum Tower Hospital Clinical College of Nanjing Medical University, Nanjing, China; b Department of Respiratory and Critical Care Medicine, Nanjing Drum Tower Hospital, Affiliated Hospital of Medical School, Nanjing University, Nanjing, China.

**Keywords:** DLCO, FVC, HRCT, IPF, pirfenidone

## Abstract

Idiopathic pulmonary fibrosis (IPF) is a debilitating condition, with a life expectancy of 2 to 5 years after diagnosis. Pirfenidone is a drug that has been shown to reduce the decline in forced vital capacity (FVC). We sought to identify whether different patterns on high-resolution computed tomography (HRCT) have different clinical effects through a retrospective comparison of baseline values and changes in pulmonary function tests (PFTs) after treatment with pirfenidone. We retrospectively analyzed data from IPF patients treated with pirfenidone at Nanjing Drum Tower Hospital in Jiangsu Province, China. According to the HRCT pattern, the patients were divided into usual interstitial pneumonitis (UIP) and possible UIP groups. Baseline clinical characteristics and changes every 6 months in the PFTs during the follow-up period were compared between the 2 groups. A total of 65 consecutive patients were enrolled. According to the HRCT pattern, patients were clustered into the UIP group (n = 46) and possible UIP group (n = 19). No difference was observed in the baseline PFTs ratio between the 2 groups. The FVC values of the 2 groups were not significantly different at the initial treatment and at 6 and 12 months after pirfenidone treatment (*P* = .081, 0.099, and 0.236, respectively). The improvement in % diffusion capacity of the lung for carbon monoxide (%DLCO) was higher in the possible UIP group after 6 and 12 months of pirfenidone treatment (*P* = .149, 0.026, and 0.025, respectively). The annual decrease in FVC was not significantly different between the 2 groups, and the annual decrease in %DLCO in the UIP group was significantly higher than that in patients with the possible UIP type (−7.767 ± 12.797 vs 0.342 ± 20.358, *P* < .05). These results indicate that patients with IPF with a possible UIP pattern on HRCT showed indications of a good response to pirfenidone.

## 1. Introduction

Idiopathic pulmonary fibrosis (IPF) is a chronic progressive fibrotic interstitial pneumonia that occurs mainly in the elderly population. Once diagnosed, most patients experience increasing dyspnea and loss of pulmonary function and have a pessimistic prognosis. High-resolution computed tomography (HRCT) is characterized by the presence of subpleural and basal predominance, reticular opacities, and honeycombing with or without traction bronciectasis.^[1,2]^ IPF is associated with a histopathological pattern of usual interstitial pneumonitis (UIP).^[3]^ On HRCT, UIP is characterized by honeycombing, which is critical for definite diagnosis, reticular opacities, traction bronchiectasis, and the absence of other patterns (e.g., consolidation) suggestive of other diseases.^[2,4]^ Like other idiopathic interstitial lung disorders, UIP is not a specific diagnosis. Actually, it is a radiographic and pathologic pattern.^[5]^ Most importantly, CT findings alone can now be used to diagnose UIP, without the need for tissue confirmation. The new guidelines list 3 levels of HRCT-based certainty for the UIP pattern: UIP, possible UIP, and inconsistent with UIP.^[6]^ HRCT is a mandatory tool for distinguishing between the 3 subtypes. The imaging abnormalities of IPF on HRCT are not only for diagnosis^[2,7]^ but also for staging its severity and prognosis. Pirfenidone is approved worldwide for the treatment of IPF because of its ability to slow functional decline and disease progression.^[8,9]^

The clinical course and outcomes of patients is highly variable and unpredictable. Some patients remain stable for extended periods, and some exhibit a progressive decline in respiratory status at variable intervals, whereas others suffer acute exacerbations that have a high mortality rate.^[10]^ The heterogeneous nature of IPF makes it difficult to predict its clinical effect in individual patients, and this heterogeneity could also conceivably affect treatment response. However, in our clinic we found that different subtypes responded differently to drug reactions. CT findings have been reported to have substantial diagnostic value in IPF. Previous studies have indicated that the degree of fibrosis visually assessed on CT images is a strong independent predictor of survival in patients with IPF.^[11–13]^ The aim of this retrospective study was to assess the effects of pirfenidone treatment on CT findings in IPF. We hypothesized that the CT pattern could detect the effect of pirfenidone in patients with IPF. To date, there is little information regarding whether the response to pirfenidone is influenced by the pattern observed on HRCT.

## 2. Methods

The study protocol was approved by the Ethics Committee of the Medical School of Nanjing University, and the requirement for written informed consent was waived because of the retrospective nature of the study. IPF was diagnosed by physicians according to the 2011 American Thoracic Society (ATS)/European Respiratory Society (ERS) consensus statement.^[1]^ Patients were selected based on the following criteria: patients who were followed up in our hospital, continued pirfenidone treatment for at least 1 year, underwent CT examination at baseline, and baseline percent predicted forced vital capacity (FVC) > 50% and < 90%, percent predicted diffusing capacity of the lung for carbon monoxide > 30% and < 90%. A total of 65 patients were enrolled in this study. All the patients underwent baseline pulmonary function tests (PFTs) and HRCT. According to the HRCT pattern, patients were clustered into the UIP group and the possible UIP group. PFTs were performed at 6-month intervals after treatment with pirfenidone. This was followed up for at least 1 year. The results at up to 12 months after pirfenidone initiation were analyzed, and the patients’ characteristics are shown in Table 1.

**Table 1:** Demographic, clinical data and pulmonary function data of the study population, UIP patients (n = 46) and possible UIP patients (n = 19). Values are presented as absolute number of patients (%) or means ± SD.

	UIP (n = 46)	Possible UIP (n = 19)	*P* value
Male, n (%)	44 (95.6)	14 (77.8)	.06
Age at diagnosis, yr	66.63	64.3	.312
Smoking History-pack, yr	13.26	1.316	.011
PFTs			
FVC at diagnosis, L	2.601 ± 0.622	2.286 ± 0.721	.081
DLCO at diagnosis, %pred	52.809 ± 19.060	60.776 ± 60.776	.149
FVC at 6 mo, L	2.600 ± 0.583	2.306 ± 0.764	.099
DLCO at 6 mo, %pred	48.507 ± 17.970	60.200 ± 20.711	.026
FVC at 12 mo – L	2.568 ± 0.636	2.340 ± 0.838	.236
DLCO at 12mo, %pred	46.042 ± 16.844	57.761 ± 21.443	.025

Baseline characteristics analysis showed that there was no difference in the age of onset between the UIP type and possible UIP type. Male sex was more common in the UIP group than in the possible UIP group, but the difference was not statistically significant. The number of packs smoked per year was also higher in the UIP group.

DLCO = diffusion capacity of lung for carbon monoxide, FVC = forced vital capacity, UIP = usual interstitial pneumonitis.

### 2.1. Statistical analysis

All analyses were performed using SPSS statistical software version 22. Continuous data are expressed as median ± SD. Qualitative data were expressed as numbers (percentages), and differences between groups were examined using the χ^2^ test. Differences between groups were calculated using the 2-tailed Student *t* test. The chi-square test or Fisher’s exact test was used, as appropriate, to compare proportions. *P* value less than .05 was considered a significant difference.

## 4. Results

Sixty-five patients from June 2017 to October 2018, a total of 65 IPF patients were included in this study. Among the 65 IPF cases, 46 were diagnosed with a UIP pattern according to HRCT, and the others were diagnosed with a possible UIP pattern. The baseline patient demographics and clinical characteristics are summarized in Table 1. The smoking history of IPF patients with a UIP pattern was significantly longer (13.26 vs 1.316 pack-years, *P* = .011). There were no significant imbalances in baseline PFTs between the 2 study groups (Table 1).

**Figure 1.**
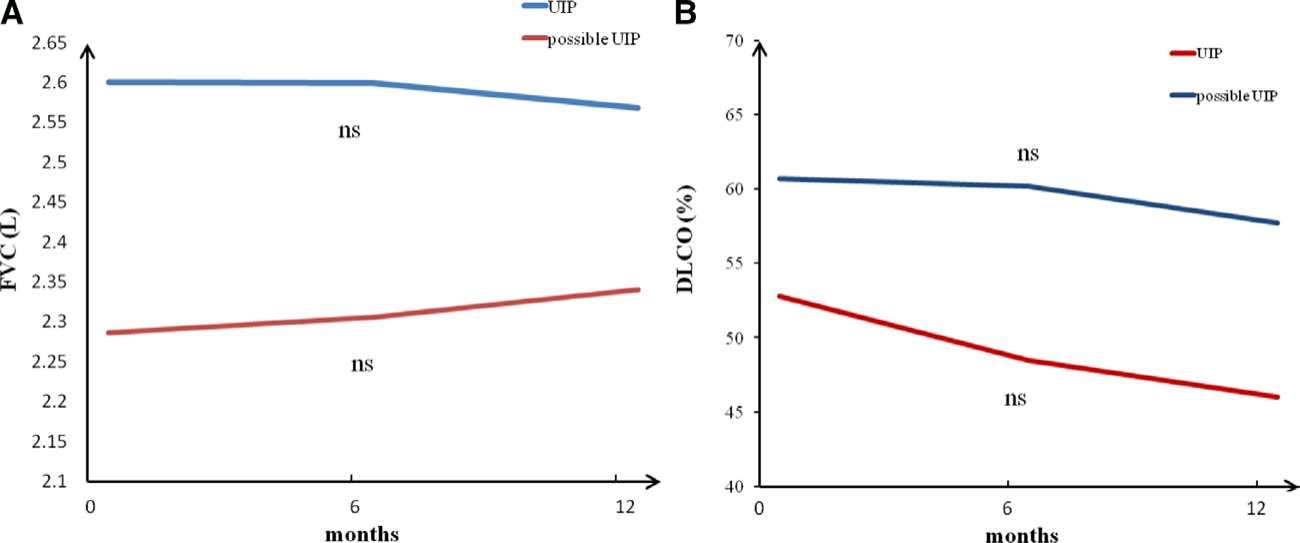
Changes of FVC (L) and DLCO% 6 and 12 months after pirfenidone treatment. (A) The absolute value of FVC in the UIP group was slightly higher at baseline and decreased at 6 and 12 months after pirfenidone treatment, but the difference was not statistically significant compared with baseline (*P* = .958). In the possible UIP group, there was an increase at 6 months and 12 months of pirfenidone treatment, butthe difference was not statistically significant (*P* = .977). (B) In the UIP group, DLCO (%) decreased at 6 and 12 months after pirfenidone treatment, but the difference was not statistically significant (*P* = .195). In the possible UIP group, there was a decrease at 6 and 12 months of pirfenidone treatment, but the difference was not statistically significant (*P* = .899). DLCO = diffusion capacity of lung for carbon monoxide, FVC = forced vital capacity, UIP = usual interstitial pneumonitis.

The absolute value of FVC in the UIP group was slightly higher at baseline and decreased at 6 and 12 months after pirfenidone treatment, but the difference was not statistically significant compared with baseline (*P* = .958). In the possible UIP group, there was an increase at 6 and 12 months of pirfenidone treatment, but it did not reach statistical significance (*P* = .977) (Fig. 1A). In the UIP group, DLCO (%) decreased at 6 and 12 months after pirfenidone treatment, but the difference was not statistically significant (*P* = .195). In the possible UIP group, there was a decrease at 6 and 12 months of pirfenidone treatment, but the difference was not statistically significant (*P* = .899) (Fig. 1B).

**Figure 2.**
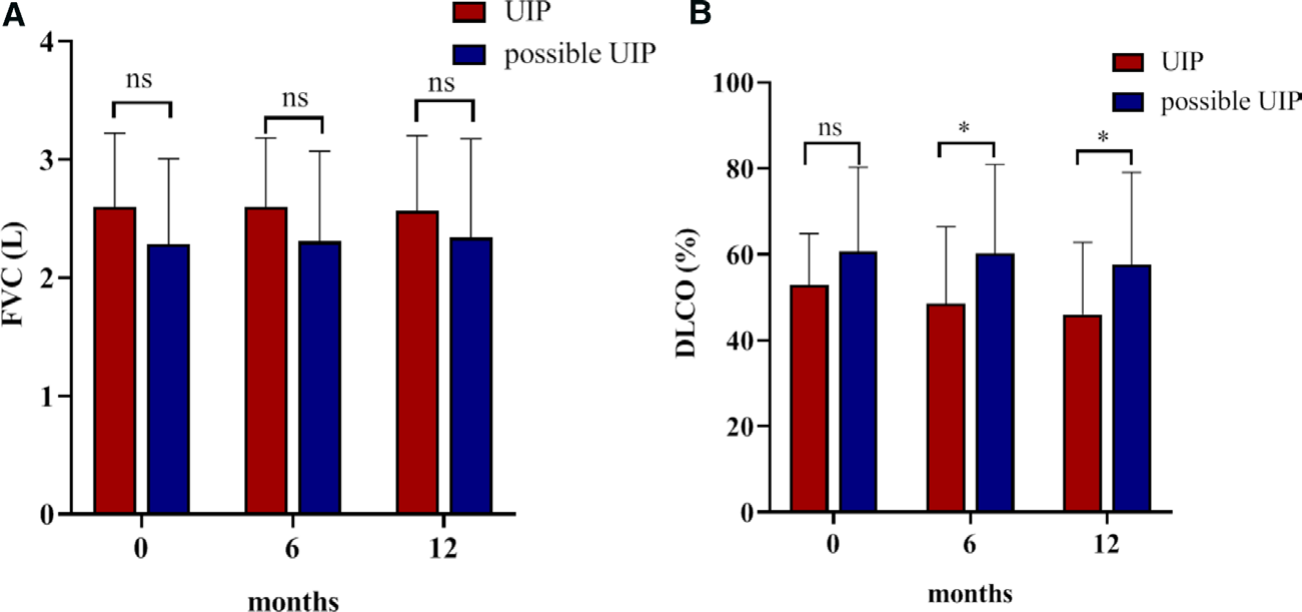
Changes of FVC (L) and DLCO% at 6 monthly intervals after pirfenidone treatment were compared between the 2 groups. (A) For changes in FVC, pirfenidone therapy offers protection against the rate of decline, which is similar between the UIP and possible UIP groups. FVC values of the 2 groups were not significantly different at the initial treatment and at 6 and 12 months after pirfenidone treatment (*P* values were .081, .099, and .236, respectively). (B) The improvement in DLCO (%) was more obvious in the possible UIP group after 6 and 12 months of pirfenidone treatment (*P* values were .149, .026, and .025, respectively). These results showed that the DLCO value of the possible UIP group was significantly higher than that of the UIP group after pirfenidone treatment. DLCO = diffusion capacity of lung for carbon monoxide, FVC = forced vital capacity, UIP = usual interstitial pneumonitis.

For changes in FVC, pirfenidone therapy offers protection against the rate of decline, which is similar between the UIP and possible UIP groups. FVC values of the 2 groups were not significantly different at the initial treatment and at 6 and 12 months after pirfenidone treatment (*P* values were .081, .099, and .236, respectively) (Fig. 2A). The improvement in %DLCO was more obvious in the possible UIP group after 6 and 12 months of pirfenidone treatment (*P* values were .149, .026, and .025, respectively (Fig. 2B). These results showed that the %DLCO value of the possible UIP group was significantly higher than that of the UIP group after pirfenidone treatment.

**Figure 3.**
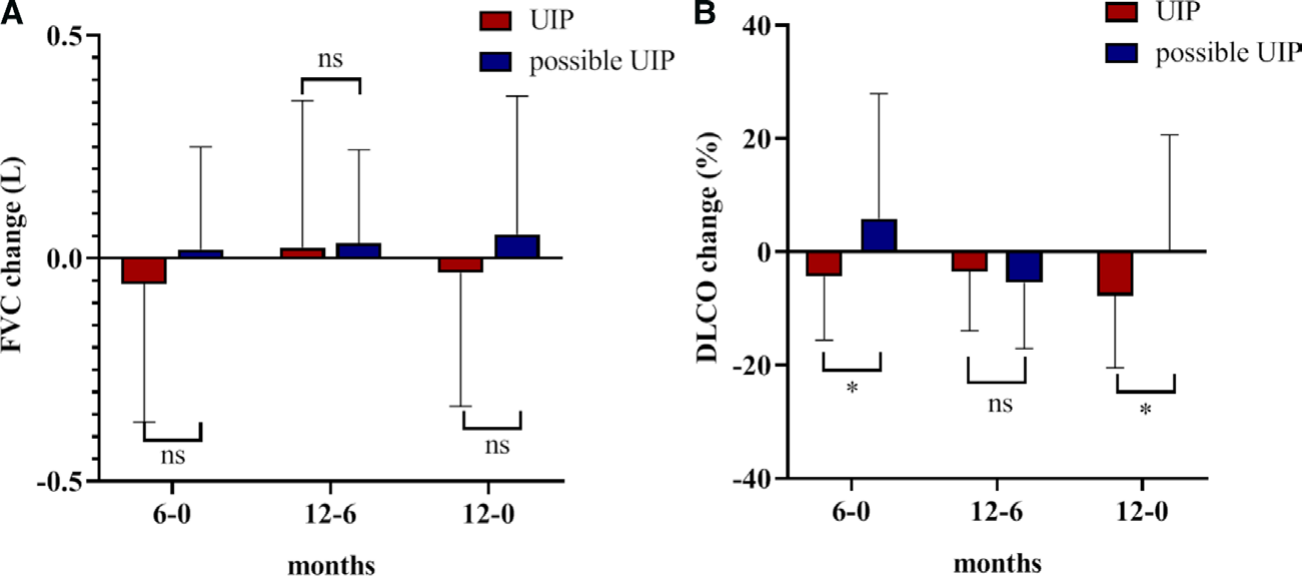
Compare the relative changes of FVC (L) and DLCO% in 6 and 12 months after pirfenidone treatment between the 2 groups. (A) After 1 year of pirfenidone treatment, changes in forced vital capacity (ΔFVC) decreased in the UIP group, especially in the first half year, while it maintained an upward trend in the possible UIP group, and the decrease in ΔFVC between the 2 groups did not reach statistical significance. (B) Changes in the %diffusion capacity of the lung for carbon monoxide (Δ%DLCO) continued to decline in the UIP group, while the level remained constant in the possible UIP group, showing an upward trend in the first half year and a downward trend in the second half year. DLCO = diffusion capacity of lung for carbon monoxide, FVC = forced vital capacity, UIP = usual interstitial pneumonitis.

After 1 year of pirfenidone treatment, relative changes of FVC(L)and DLCO% at 6 monthly intervals after pirfenidone treatment were compared between the 2 groups (showed in Table 2). The changes in forced vital capacity (ΔFVC) decreased in the UIP group, especially in the first half year, while it maintained an upward trend in the possible UIP group, and the decrease in FVC between the 2 groups did not reach statistical significance (Fig. 3A). Changes in the %diffusion capacity of the lung for carbon monoxide (Δ%DLCO) continued to decline in the UIP group, while the level remained constant in the possible UIP group, showing an upward trend in the first half year and a downward trend in the second half year (shown in Fig. 3B).

**Table 2:** Relative changes of FVC(L)and DLCO% at 6 monthly intervals after pirfenidone treatment were compared between the 2 groups.

	UIP (n = 46)	possible UIP (n = 18)	*P* value
FVC change (6–0), L	−0.0572 ± 0.310	0.020 ± 0.231	.332
DLCO change (6–0), %pred	−4.302 ± 11.385	5.821 ± 22.198	.018
FVC change (12–6), L	0.0243 ± 0.332	0.034 ± 0.205	.910
DLCO change (12–6), %pred	−3.465 ± 10.515	−5.479 ± 11.638	.499
FVC change (12–0), L	−0.0328 ± 0.303	0.054 ± 0.318	.306
DLCO change (12–0), %pred	−7.767 ± 12.797	0.342 ± 20.358	.050

DLCO = diffusion capacity of lung for carbon monoxide, FVC = forced vital capacity, UIP = usual interstitial pneumonitis.

## 5. Discussion

Idiopathic pulmonary fibrosis (IPF) is a devastating disease of unknown etiology that is limited to the lungs. The 5-year survival rate of patients with IPF is < 30%.^[1,2]^ For years, the only effective treatment available has been lung transplantation. However, only a small number of patients can benefit from it (resulting in scarcity of lung resources, post-transplant infection, etc.).

Owing to the poor prognosis of patients with IPF, 2 drugs (nintedanib and pirfenidone) have been shown to slow disease progression, reduce the decline in forced vital capacity, and increase longevity.^[9,14,15]^ Pirfenidone is an anti-fibrotic drug and the first novel agent with proven clinical efficacy in the treatment of IPF. In the ASCEND trial, pirfenidone significantly improved progression-free survival in patients with IPF and slowed the decline in FVC at 52 weeks. Pirfenidone was approved by the Food and Drug Administration in 2014 for patients with IPF.^[9]^

In the real world, we found that the efficacy of pirfenidone varies widely among individuals, and some patients discontinued treatment because of side effects (gastrointestinal reactions or hepatic insufficiency). Identifying key biomarkers to guide clinical treatment remains a substantial challenge in IPF patients. We speculate that there is a group of patients who would benefit more from pirfenidone treatment, the so-called drug advantage population, for which clinicians are more confident in prescribing.

To date, some research has been conducted in this area. Biondini et al^[16]^ found that patients with rapidly progressive IPF could benefit more from pirfenidone treatment. Basing on the annual FVC decline of patients who did not take pirfenidone for 1 year after diagnosis, they classified IPF patients into 2 groups, stable or slowly progressive type (FVC% pred. ≤10%), and rapidly progressive (FVC% predicted > 10%). After taking pirfenidone for 2 years, changes in lung function in the 2 groups were compared. This study confirmed that pirfenidone treatment significantly reduced the rate of FVC decline in patients with IPF, an effect that was significantly more pronounced in patients with rapidly progressive IPF.

However, this study has certain limitations. First, the patients with rapid progression type were prone to statistical differences; second, in the real world, physicians cannot immediately determine the type of newly diagnosed IPF patients. This type is rapidly progressive or stable and needs to be observed for 1 year before treatment, which delays the patient’s condition. Therefore, the research conclusions of Biondini have little clinical significance, and they are not widely used in clinical practice.

HRCT plays an important role in the diagnosis, follow-up, and prognosis of IPF patients. The latest ATS/ERS guidelines recommend that if HRCT is typical and can be directly diagnosed without further pathology. A previous study showed that CT analysis of subpleural fibrosis had an impact on the prognosis of IPF.^[17,18]^ We hypothesized that different types can reflect the efficacy of pirfenidone in patients with IPF. We divided patients with IPF into UIP patterns and possible UIP patterns based on HRCT before pirfenidone treatment. Our study aimed to explore which HRCT pattern of IPF patients responded better to pirfenidone treatment.

Therefore, we retrospectively examined the imaging features of 65 patients diagnosed with IPF with relevance to the effective factors for pirfenidone. FVC values of both UIP and possible UIP did not decrease significantly after pirfenidone treatment, and the treatment was effective but showed an upward trend in the possible UIP group. The %DLCO values of both UIP and possible UIP did not decrease significantly after pirfenidone treatment, suggesting that treatment could delay the decline in %DLCO, and the decrease was slower in the possible UIP group. For changes in FVC, pirfenidone therapy offers protection against the rate of decline, which is similar between the UIP and possible UIP groups. The improvement in %DLCO was more obvious in the possible UIP group after 6 and 12 months of pirfenidone treatment, which might be explained by the hypothesis that the beneficial effect was greater in IPF cases with a possible UIP pattern on HRCT.

Our study has several limitations. First, this was a retrospective, single-institution clinical study with a small study cohort. Moreover, the follow-up period in our study was not long enough. Further studies are needed to confirm these encouraging results.

## 6. Conclusion

In summary, most of these IPF patients may benefit from treatment with pirfenidone, especially in patients with a possible UIP pattern on HRCT, which might be associated with a good response to pirfenidone. To our knowledge, this is the first case series to report that among patients with IPF, the possible UIP pattern on HRCT may be associated with a good prognosis and may represent a novel, useful, and noninvasive tool to assess and predict the effect of pirfenidone treatment.

## Author contributions

**Conceptualization:** Miao Ma, Hourong Cai.

**Data curation:** Miao Ma, Min Cao.

**Formal analysis:** Yujuan Gao.

**Funding acquisition:** Hourong Cai.

**Investigation:** Xiaohua Qiu, Hanyi Jiang.

**Methodology:** Yujuan Gao.

**Supervision:** Min Cao, Xiaohua Qiu, Hanyi Jiang.

**Writing – original draft:** Miao Ma, Min Cao.

**Writing – review & editing:** Hourong Cai.
